# Assessment of Zygomaticomaxillary Suture Maturation in Patients With Unilateral Cleft Lip and Palate: Implications for Orthodontic Treatment Planning

**DOI:** 10.7759/cureus.70146

**Published:** 2024-09-25

**Authors:** Niharika Bhatia, Navaneethan R

**Affiliations:** 1 Department of Orthodontics, Saveetha Dental College and Hospitals, Saveetha Institute of Medical and Technical Sciences, Saveetha University, Chennai, IND

**Keywords:** cleft orthodontics, orthodontics, suture ossification, unilateral cleft lip, unilateral cleft lip and palate

## Abstract

Introduction

The zygomaticomaxillary suture (ZMS) is vital for craniofacial development and orthodontic treatment planning. Located at the junction of the zygomatic and maxillary bones, the ZMS undergoes significant changes during growth, affecting midfacial morphology and stability. Understanding ZMS maturation is essential for optimizing orthodontic interventions, especially in patients with cleft lip and palate (CLP), who often exhibit altered craniofacial growth patterns due to congenital deformities and surgical interventions.

Methodology

A retrospective analysis was conducted with 50 participants, including 25 patients with unilateral CLP and 25 age-matched controls. Clinical and radiographic data, including lateral cephalometric radiographs and cone-beam computed tomography (CBCT) scans, were collected to assess ZMS maturation stages. The stages, ranging from immature (stage 0) to fully mature (stage 4), were evaluated using established radiographic criteria. Statistical analyses, including Chi-square tests and logistic regression, were performed to compare the distribution of maturation stages between groups.

Results

Significant differences were observed in ZMS maturation stages between the cleft side, non-cleft side, and control group. The cleft side exhibited a higher prevalence of immature (stage 0) and early maturation (stage 1) stages compared to the control group, with mean values of 4 ± 1.2 and 5 ± 1.3, respectively. The control group showed a higher prevalence of fully mature (stage 4) sutures, with a mean value of 8 ± 1.4. The p-values for stages 0, 1, and 4 were 0.045, 0.034, and 0.039, respectively, indicating statistical significance. The findings highlight distinct maturation patterns in CLP patients, with delayed ZMS maturation on the cleft side. This suggests the need for tailored orthodontic interventions to accommodate prolonged growth phases in CLP patients. The study underscores the importance of detailed ZMS assessments for personalized treatment planning. Future research should focus on longitudinal studies to further understand CLP’s impact on craniofacial development.

Conclusion

Assessing ZMS maturation provides critical insights for optimizing orthodontic and surgical treatment outcomes in CLP patients. By identifying specific developmental delays and tailoring interventions accordingly, clinicians can improve the effectiveness and stability of treatments. This study contributes to the growing body of evidence supporting individualized treatment plans based on precise developmental assessments.

## Introduction

The zygomaticomaxillary suture (ZMS) is integral to craniofacial development and orthodontic treatment planning [1]. This suture, located at the junction of the zygomatic and maxillary bones, undergoes significant changes during growth and development, influencing midfacial morphology and stability [2]. The evaluation of ZMS maturation helps clinicians determine the optimal timing for treatments that require maxillary growth modification [1]. Studies have shown that the timing of orthodontic interventions significantly impacts treatment outcomes, making the assessment of ZMS maturation crucial for personalized treatment plans [2]. Evaluating the maturation of the ZMS is particularly important in patients with cleft lip and palate (CLP). These individuals often exhibit altered craniofacial growth patterns due to congenital deformities and the impact of surgical interventions performed early in life [3]. The presence of CLP can lead to asymmetries and deviations in normal craniofacial development, complicating orthodontic treatment planning and execution [4]. Underdevelopment of the ZMS in patients with unilateral CLP can lead to several significant anatomical and functional complications [3].

This underdevelopment primarily arises from the disrupted fusion and misalignment of the maxillary and zygomatic bones, which are integral to the facial structure [5]. The resulting asymmetry can be quite pronounced, affecting both the appearance and functionality of the midface. One of the primary effects is facial asymmetry, where the affected side of the face appears flattened or underdeveloped compared to the unaffected side [6]. This can lead to a noticeable imbalance in the facial contour, particularly in the infraorbital region. The midfacial deficiency may also extend to the dental arch, contributing to malocclusion where the teeth do not align properly. This can cause difficulties in chewing and speaking, and may necessitate orthodontic and surgical interventions to correct. Furthermore, the underdevelopment of the ZMS can affect the growth and alignment of the orbital cavity, potentially leading to ocular issues such as enophthalmos (sunken eye) or strabismus (misalignment of the eyes) [7]. This is because the zygomatic bone helps form the orbit's lateral wall and floor, and any malformation can impact the positioning and support of the eyeball. Additionally, the compromised structural integrity can lead to nasal deformities, as the maxilla plays a critical role in supporting the nasal framework [6].

Surgical correction in patients with unilateral CLP aims to address these multifaceted issues by realigning the bones, restoring symmetry, and ensuring proper function. This typically involves a multidisciplinary approach, including craniofacial surgeons, orthodontists, and speech therapists, to achieve the best possible outcomes in both appearance and functionality [5]. Understanding the intricate effects of ZMS underdevelopment is crucial for planning and executing these comprehensive treatment strategies. Research has indicated that craniofacial growth in CLP patients is often characterized by maxillary hypoplasia and altered midfacial structures [8].

As a result, assessing the ZMS maturation in these patients is vital to understanding the extent of growth deviations and tailoring treatment plans that can address these unique challenges. The ZMS maturation stages are categorized using established radiographic criteria, which classify the suture into distinct stages ranging from immature (stage A) to fully mature (stage E). Stage A represents an immature suture with no signs of ossification. Stage B indicates early maturation with initial ossification centers appearing. Stage C, or intermediate maturation, shows further ossification but incomplete fusion. Stage D, advanced maturation, is characterized by nearly complete ossification with only small gaps remaining. Finally, stage E represents a fully mature suture with complete ossification and no visible gaps. These stages are crucial for determining the developmental progress of the ZMS and are especially relevant in assessing the differences between CLP and non-CLP patients [9].

This retrospective study aims to assess the differences in ZMS maturation between patients with and without CLP, providing insights that could improve orthodontic and surgical treatment outcomes.

## Materials and methods

The study was approved by the Independent Ethics Committee of Saveetha Dental College and Hospital, affiliated with Saveetha Institute of Medical and Technical Sciences (IHEC/SDC/ORTHO-2107/24/165). This retrospective study involved a total of 50 participants, comprising 25 patients with unilateral CLP and 25 age-matched controls without CLP. The participants were selected from past medical records of patients who had received orthodontic treatment at a tertiary care center and mostly included children of the Indian population. Inclusion criteria for the CLP group included patients aged 10-18 years with surgically repaired unilateral CLP and no history of orthodontic treatment, while the study excluded participants with bilateral clefts of the lip and palate and incomplete clefts of the palate. The control group consisted of individuals with no craniofacial anomalies or previous orthodontic treatment. Informed consent was obtained from all participants or their guardians for the regular orthodontic treatment.

Cone-beam computed tomography (CBCT) images were obtained for each patient as a general investigation tool, and no additional radiographs were obtained specifically for this study. Clinical and radiographic data were collected for all participants. Lateral cephalometric radiographs were extracted from the CBCT scan using Dolphin 3D imaging software, and both were utilized to assess the ZMS maturation stages. All CBCTs were taken following standardized protocols to ensure consistency and accuracy. Demographic data, including age, sex, and medical history, were recorded and tabulated as shown in Table 1.

**Table 1:** Characteristics of cleft lip and palate (CLP) and control groups.

Demographic Characteristics	Cleft Lip and Palate Group (n = 25)	Control Group (n = 25)	Total (n = 50)
Age (Years)			
10-12	6	5	11
13-15	10	12	22
16-18	9	8	17
Mean Age	14.1	14.2	14.1
Sex			
Male	16	15	31
Female	9	10	19

**Figure 1:**
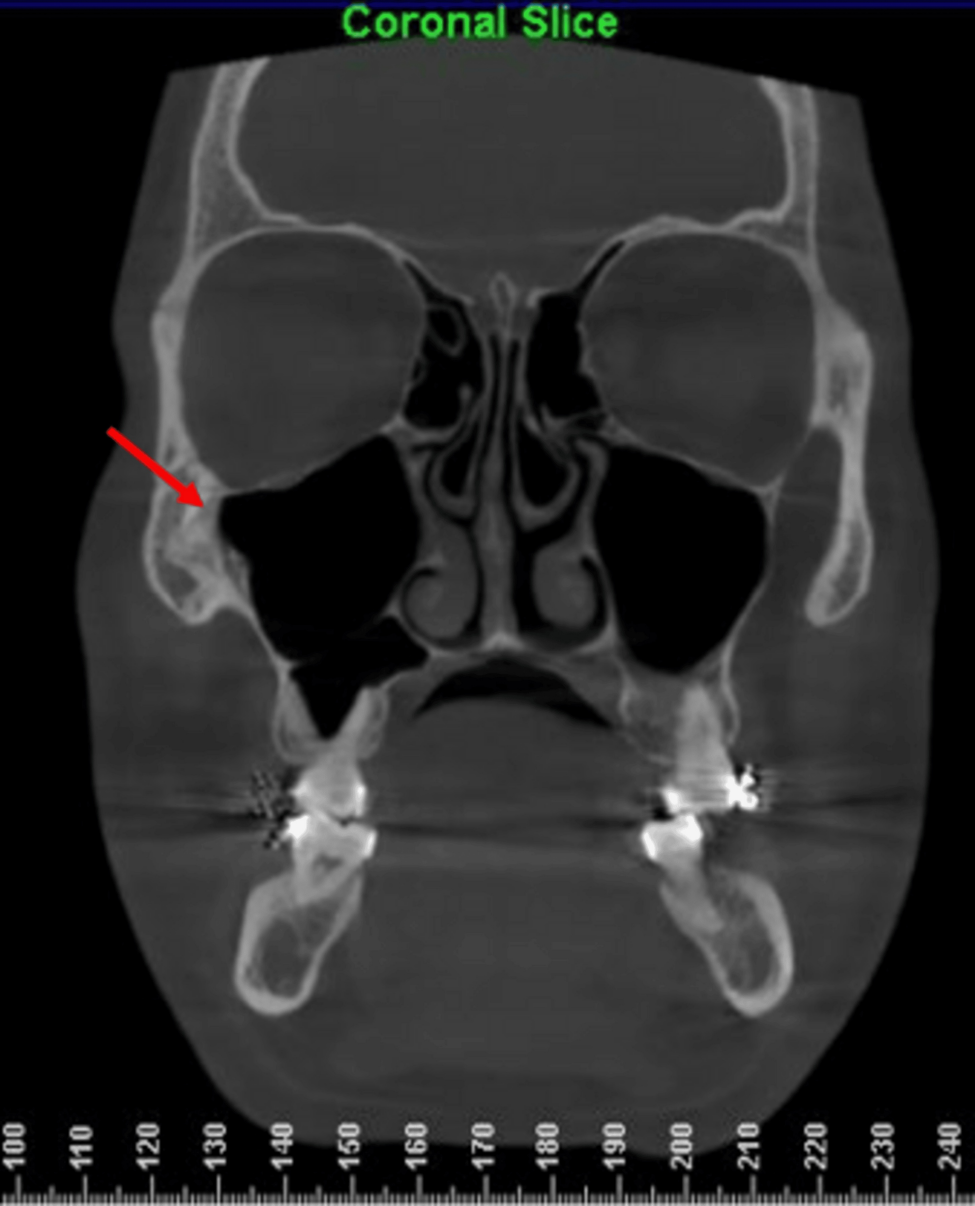
Coronal slice on a CBCT showing the exact location of the zygomaticomaxillary suture. The red arrows indicate the exact location of the suture in all coronal sections. CBCT: Cone-beam computed tomography.

**Figure 2:**
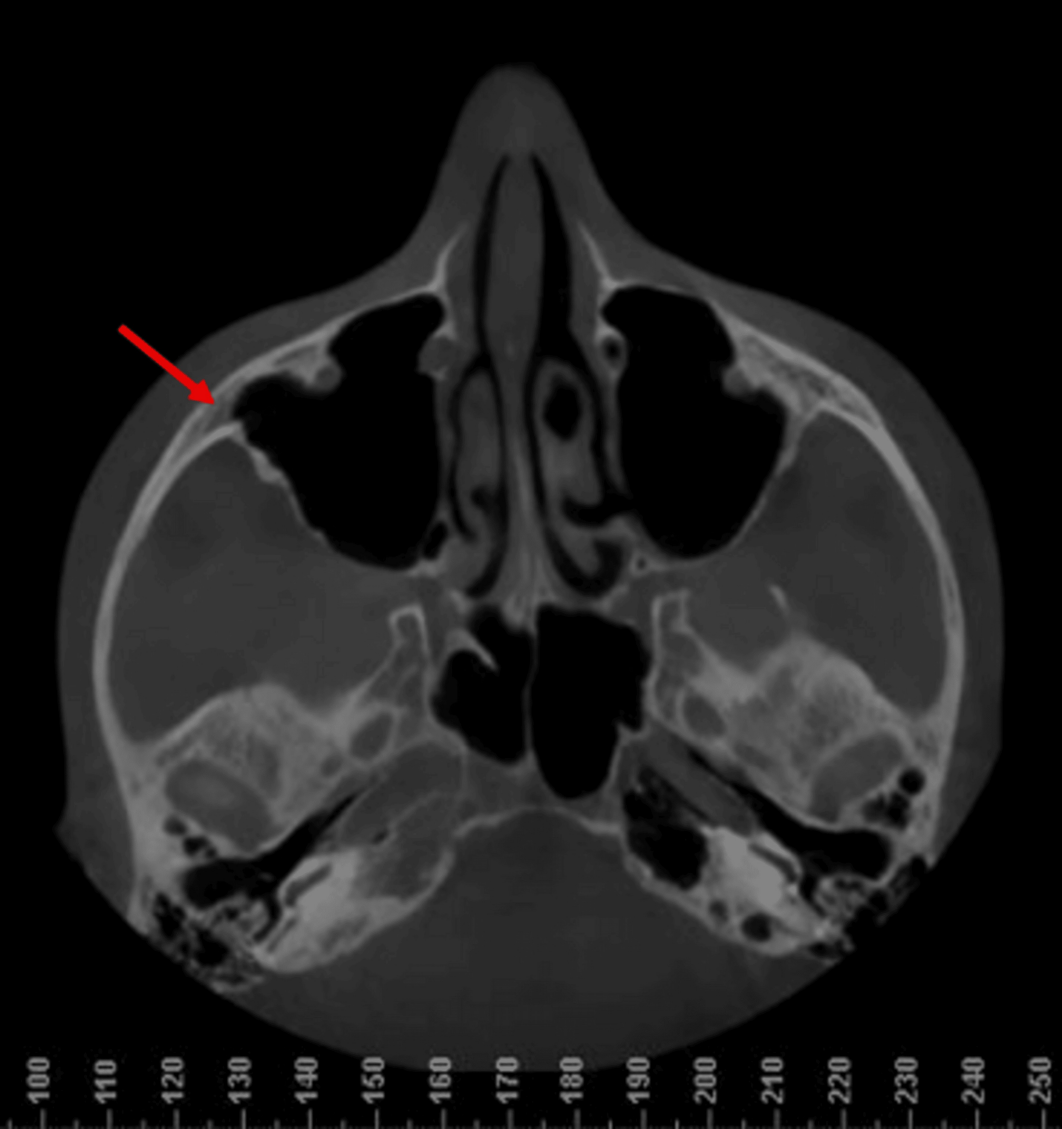
Axial slice on a CBCT indicating the exact location of the zygomaticomaxillary suture (red arrows). CBCT: Cone-beam computed tomography.

All radiographic images were evaluated by two independent orthodontists to ensure reliability and minimize observer bias. The CBCT section showing the ZMS as identified on the Dolphin 3D imaging software is shown in Figures 1-2.

The maturation of the ZMS was evaluated using established radiographic criteria proposed by Angelieri F et al. [9]. This method categorizes the suture into five distinct stages, each reflecting a different level of ossification and maturity. In the initial stage, known as Stage A, there is a uniform high-density sutural line, with no or little interdigitation. As the suture progresses to Stage B, there is a scalloped appearance of the high-density sutural line. These centers are small and scattered, marking the beginning of the ossification process. In Stage C, there are two parallel, scalloped, high-density lines, separated in some areas by small low-density spaces. Nearly complete ossification occurs in Stage D, where fusion is seen in the inferior portion of the suture. Finally, in Stage E (fully mature), the suture exhibits complete ossification with no visible gaps, indicating full maturity. To determine the maturation stage for each participant, CBCT scans and lateral cephalograms were meticulously examined. Two experienced radiologists independently reviewed the imaging data of all the selected CBCTs, to assign a maturation stage. Inter-examiner agreement was assessed using Cohen’s kappa coefficient, ensuring a high level of consistency between the evaluators. This statistical measure was chosen because it provides a robust method for assessing the reliability of categorical data, particularly when two raters are involved. The collected data were analyzed using SPSS software (version 25.0). Descriptive statistics were calculated to summarize the demographic variables and the distribution of ZMS maturation stages among the participants. This initial analysis provided a comprehensive overview of the sample characteristics and facilitated subsequent comparative analyses. To compare the distribution of ZMS maturation stages between the unilateral CLP group and the control group, Chi-square tests were employed. This non-parametric test was chosen for its suitability in examining the association between categorical variables, allowing for the identification of significant differences in maturation stage distributions between the two groups. A p-value of less than 0.05 was considered statistically significant for all tests.

## Results

The findings of the ZMS maturation stages in patients with unilateral CLP and control participants are summarized in Table 2. Mean values and p-values are included to highlight the statistical significance of the differences observed between the two groups.

**Table 2:** Zygomaticomaxillary suture maturation stages. Mean values and p-values are included to highlight the statistical significance of the differences observed between the two groups. The statistical test used to analyze the p-values is one-way analysis of variance (ANOVA). * in the table denotes a statistically significant value.

Maturation stage	Cleft-side (n=25)	Non cleft side (n=25)	Control group (n=25)	Mean ± SD (cleft side)	Mean ± SD (non-cleft side)	Mean ± SD (control group)	P-value
Stage A (Immature)	4	3	1	4 ± 1.2	3 ± 1.0	1 ± 0.9	0.045*
Stage B (Early maturation)	5	4	3	5 ± 1.3	4 ± 1.1	3 ± 1.0	0.034*
Stage C ( Intermediate maturation)	8	7	9	8 ± 1.5	7± 1.4	9 ± 1.3	0.067
Stage D (Advanced maturation)	5	6	5	5 ± 1.1	6 ± 1.2	5 ± 1.3	0.052
Stage E (Fully maturation)	3	5	7	3 ± 1.0	5 ± 1.3	7 ± 1.4	0.039*
Total Sample size	25	25	25				

The results obtained, as shown in Table 2, indicate the varying degrees of ZMS maturation in patients with unilateral CLP compared to the control group. For Stage A, the mean values were 4 ± 1.2 for the cleft side, 3 ± 1.0 for the non-cleft side, and 1 ± 0.9 for the control group. The p-value for this stage was 0.045, indicating a statistically significant difference between the groups. This suggests that the cleft side exhibits a higher degree of immaturity compared to both the non-cleft side and the control group. In Stage B, the mean values increased to 5 ± 1.3 for the cleft side, 4 ± 1.1 for the non-cleft side, and 3 ± 1.0 for the control group. The p-value of 0.034 for this stage also indicates a statistically significant difference, with the cleft side showing more early maturation than the control group. This trend continues to reflect the delayed or disrupted maturation process on the cleft side. At Stage C, the mean values were 8 ± 1.5 for the cleft side, 7 ± 1.4 for the non-cleft side, and 9 ± 1.3 for the control group. The p-value of 0.067 for this stage indicates that the differences observed are not statistically significant, suggesting that the intermediate maturation stage is more uniformly distributed among the groups. In Stage D, the mean values were 5 ± 1.1 for the cleft side, 6 ± 1.2 for the non-cleft side, and 5 ± 1.3 for the control group. With a p-value of 0.052, this stage also does not show a statistically significant difference, indicating that the maturation process in this advanced stage is relatively comparable across the groups. Finally, at Stage E, the mean values were 3 ± 1.0 for the cleft side, 5 ± 1.3 for the non-cleft side, and 7 ± 1.4 for the control group. The p-value for this stage was 0.039, highlighting a significant difference. This indicates that the cleft side lags in achieving full maturity compared to both the non-cleft side and the control group. These summarized findings illustrate significant differences in the maturation stages of the ZMS between the cleft side and the control group at various stages, particularly at stages A, B, and E. These p-values underscore the statistical significance of these differences, highlighting the critical need for tailored orthodontic interventions based on the specific maturation stage of each patient. Such insights are vital for optimizing treatment strategies and improving clinical outcomes for patients with unilateral cleft lip and palate.

## Discussion

**Figure 3:**
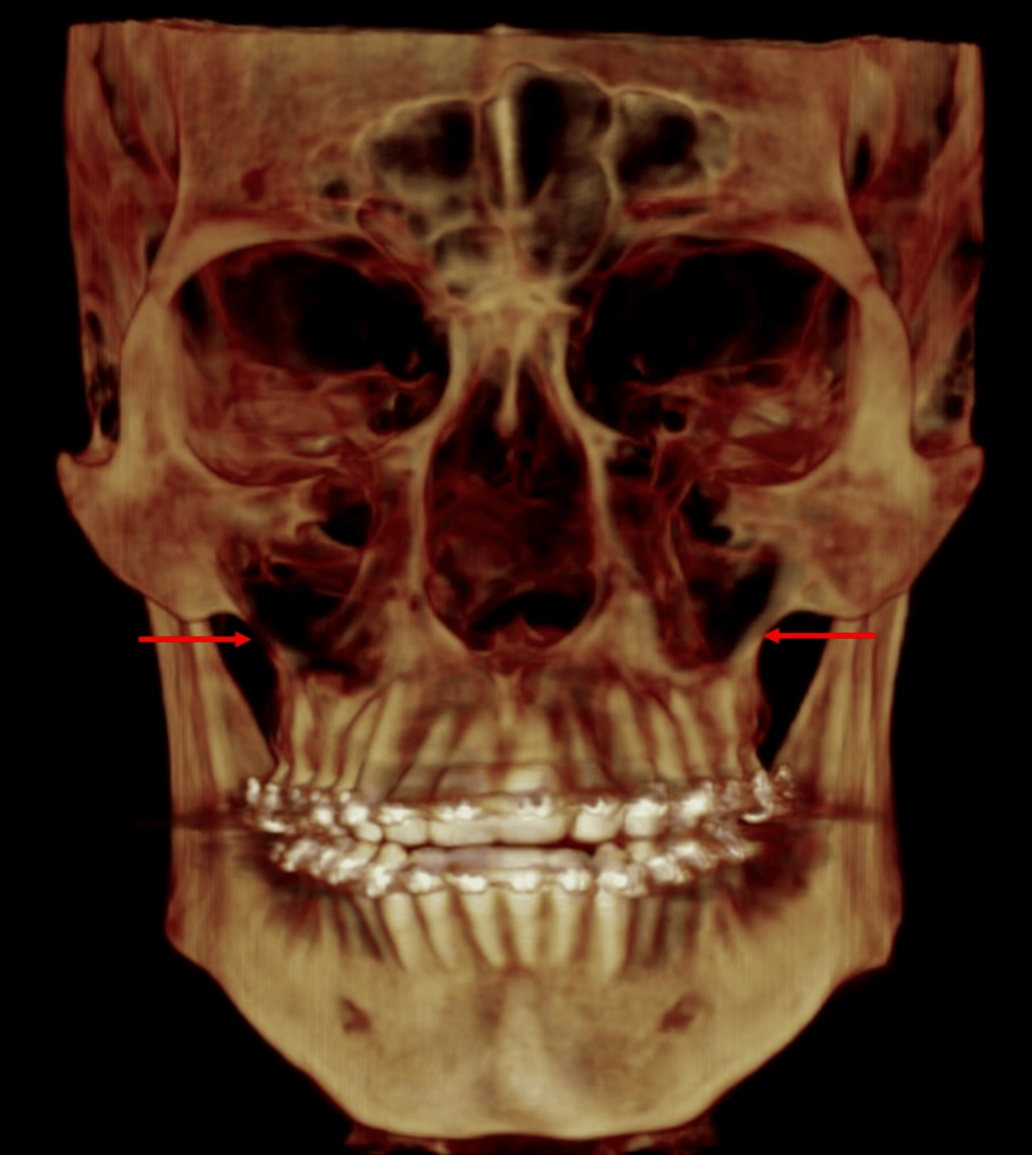
A cone beam computed tomography (CBCT) image showing the exact location of the zygomaticomaxillary suture in a 3D volume as imaged on the Dolphin 3D software. The red arrow indicates the exact location of the zygomaticomaxillary suture (ZMS). Image credits: Niharika Bhatia.

The ZMS is a craniofacial anatomical feature where the zygomatic bone and the maxilla meet. Located on the lateral aspect of the face, this suture plays a crucial role in the structural integrity and aesthetic contour of the midface (Figure 3) [10].

It contributes to the formation of the infraorbital rim, which supports the eye and the surrounding soft tissues. The ZMS is significant for various functions, including facial symmetry, mastication, and protection of the ocular and nasal cavities. It also serves as an important landmark for surgeons during facial reconstruction and cosmetic procedures [11]. In patients with unilateral CLP, the ZMS often exhibits notable differences compared to individuals without this congenital anomaly [12]. The clefting process can lead to asymmetry and malalignment of the maxillary and zygomatic bones, resulting in an altered or disrupted suture line. This disruption can affect facial growth and development, leading to challenges in occlusion, mastication, and aesthetic appearance [11].

Understanding these anatomical differences is essential for planning effective surgical repairs and optimizing functional and aesthetic outcomes for patients with unilateral CLP [1, 3]. The maturation of the ZMS is a crucial aspect of craniofacial development, significantly influencing orthodontic treatment planning, especially in patients with CLP [9]. This study aimed to evaluate the differences in ZMS maturation between patients with unilateral CLP and individuals without CLP, providing insights that could enhance treatment strategies and outcomes. Our findings reveal distinct maturation patterns between the cleft side, non-cleft side, and control group, underscoring the impact of CLP on craniofacial growth and development.

Our results demonstrate significant differences in the early and late stages of ZMS maturation between the cleft side and the control group. Specifically, the cleft side exhibited a higher prevalence of immature (stage A) and early maturation (stage B) stages compared to the control group. These findings are consistent with previous studies that have reported delayed craniofacial development in CLP patients, likely due to the congenital anomalies and subsequent surgical interventions affecting normal growth patterns [13-18]. The higher prevalence of immature sutures on the cleft side suggests that these patients may require prolonged or differently timed orthodontic interventions to achieve optimal outcomes [17, 18].

In contrast, the control group showed a higher prevalence of fully mature (stage E) ZMS compared to both the cleft and non-cleft sides of CLP patients. This indicates that individuals without CLP generally reach full suture maturation earlier, supporting the notion that CLP significantly disrupts normal craniofacial development [15, 19]. The delayed maturation on the cleft side might necessitate specific orthodontic strategies to accommodate the prolonged growth phase, thereby improving treatment efficacy and stability. The differences in ZMS maturation between the cleft and non-cleft sides within the same individuals also highlight the asymmetric nature of craniofacial growth in CLP patients. This asymmetry poses additional challenges in treatment planning, as orthodontists must account for the varying maturation stages within the same patient. Previous research has emphasized the need for individualized treatment plans tailored to the specific growth patterns of each patient, particularly in complex cases like CLP. Our study further supports this approach, suggesting that a detailed assessment of ZMS maturation can guide more effective and personalized treatment strategies.

The methodology of using established radiographic criteria to categorize ZMS maturation stages provided a robust framework for our analysis. The stages, ranging from immature (stage A) to fully mature (stage E), allowed for a detailed evaluation of the suture's development. This approach aligns with the work of Okamoto T et al. [19], who demonstrated the utility of ultrasonic imaging criteria in assessing craniofacial sutures. By employing these criteria, we ensured that our findings were both reliable and comparable to existing literature, enhancing the validity of our results. Our study underscores the critical role of ZMS maturation in orthodontic treatment planning for CLP patients. The significant differences in maturation stages between the cleft side, non-cleft side, and control group highlight the need for tailored interventions based on detailed developmental assessments. By incorporating advanced radiographic criteria, clinicians can enhance their understanding of craniofacial growth patterns, leading to more effective and personalized treatment strategies. Future research should focus on longitudinal studies to further elucidate the impact of CLP on craniofacial development and refine predictive models for clinical use.

While this study provides valuable insights into the differences in ZMS maturation between patients with unilateral CLP and control participants, several limitations should be noted. First, the retrospective nature of the study may introduce selection bias, as the data were obtained from medical records, limiting control over the sample selection process. The relatively small sample size (n = 50) may also restrict the generalizability of the findings to larger populations or different demographic groups. Additionally, the use of cross-sectional data limits our ability to assess longitudinal changes in ZMS maturation over time, which is critical for understanding how these differences evolve throughout growth and development. Future studies should consider a longitudinal design to track the progression of suture maturation stages in CLP patients. Furthermore, while radiographic criteria were employed to evaluate ZMS maturation, inherent limitations in imaging techniques, such as variations in CBCT resolution and the subjective nature of stage classification, could influence the accuracy of the results. Lastly, this study did not account for potential confounding factors such as the timing and type of surgical intervention, which could significantly affect craniofacial growth and suture maturation. Future research should aim to control for these variables to provide a more comprehensive understanding of the maturation patterns in CLP patients.

## Conclusions

The results of this study underscore the importance of assessing ZMS maturation in patients with CLP for optimizing orthodontic and surgical treatment outcomes. The significant differences observed in ZMS maturation stages between the cleft side, non-cleft side, and control group highlight the distinct craniofacial development patterns in CLP patients. Notably, the delayed maturation on the cleft side emphasizes the necessity for tailored orthodontic interventions that consider these prolonged growth phases. By identifying specific developmental delays, clinicians can formulate individualized treatment plans that improve the effectiveness and stability of interventions. The findings suggest that incorporating detailed ZMS assessments into treatment planning could enhance patient outcomes, particularly in those with congenital craniofacial anomalies. Longitudinal studies are essential to deepen our understanding of CLP's impact on craniofacial development and to refine intervention strategies. This research contributes to the growing body of evidence advocating for personalized orthodontic care based on precise developmental assessments, ultimately aiming to improve the quality of life for patients with CLP.
